# Increased MCL-1 synthesis promotes irradiation-induced nasopharyngeal carcinoma radioresistance via regulation of the ROS/AKT loop

**DOI:** 10.1038/s41419-022-04551-z

**Published:** 2022-02-08

**Authors:** Ying-Ying Liang, Fei-Yu Niu, An-An Xu, Li-Li Jiang, Chun-shan Liu, Hui-ping Liang, Yu-Fan Huang, Xun-Fan Shao, Zhi-Wen Mo, Ya-Wei Yuan

**Affiliations:** 1 Department of Radiation Oncology, Affiliated Cancer Hospital & Institute of Guangzhou Medical University, Guangzhou, China; 2 Department of Internal Medicine, Section 3, Affiliated Cancer Hospital & Institute of Guangzhou Medical University, Guangzhou, China; 3 Guangzhou Municipal and Guangdong Provincial Key Laboratory of Protein Modification and Degradation, School of Basic Medical Science, Guangzhou Medical University, Guangzhou, China

**Keywords:** Experimental models of disease, Radiotherapy, Head and neck cancer

## Abstract

Worldwide, nasopharyngeal carcinoma (NPC) is a rare head and neck cancer; however, it is a common malignancy in southern China. Radiotherapy is the most important treatment strategy for NPC. However, although radiotherapy is a strong tool to kill cancer cells, paradoxically it also promotes aggressive phenotypes. Therefore, we mimicked the treatment process in NPC cells in vitro. Upon exposure to radiation, a subpopulation of NPC cells gradually developed resistance to radiation and displayed cancer stem-cell characteristics. Radiation-induced stemness largely depends on the accumulation of the antiapoptotic myeloid cell leukemia 1 (MCL-1) protein. Upregulated MCL-1 levels were caused by increased stability and more importantly, enhanced protein synthesis. We showed that repeated ionizing radiation resulted in persistently enhanced reactive oxygen species (ROS) production at a higher basal level, further promoting protein kinase B (AKT) signaling activation. Intracellular ROS and AKT activation form a positive feedback loop in the process of MCL-1 protein synthesis, which in turn induces stemness and radioresistance. AKT/MCL-1 axis inhibition attenuated radiation-induced resistance, providing a potential target to reverse radiation therapy-induced radioresistance.

## Introduction

Nasopharyngeal carcinoma (NPC) is a kind of head and neck cancer arising from the nasopharyngeal epithelium [1]. NPC has a unique pattern of geographical distribution, and newly diagnosed cases are most common in South China and Southeast Asia [2, 3]. Nonkeratinizing undifferentiated NPC cells are highly sensitive to radiation therapy (RT) [4]; therefore, RT (with or without chemotherapy) is the standard curative treatment for NPC [5]. Ionizing radiation (IR) induces cell death via direct and indirect effects. Direct effects include chromosomal aberrations, such as nuclear DNA damage and mutagenesis. IR also induces indirect damage through reactive oxygen species (ROS) produced by water radiolysis [6]. However, low levels of ROS also promote secondary cancer or tumor progression [7, 8]. Cancer cells that survive IR treatment display more aggressive phenotypes (including epithelial to mesenchymal transition (EMT)) [9, 10] and enriches the fraction of cancer stem cells [11–13]. Both are closely related to metastasis and therapeutic resistance.

Cancer stem cells (CSCs) are a small subpopulation of cells displaying properties such as self-renewal, differentiation, tumorigenic capabilities, and increased resistance to treatment [14, 15]. In many cancers, the existence of CSCs has been found to interfere with treatment success [16]. Radiation induces CSC generation or enriches their subpopulation from non-stem cancer cells [17, 18]. Intracellular ROS levels are critical to maintain CSC properties in breast cancer and Head and Neck Cancer cells [19]. In contrast to cancer cells that obtain energy from glycolysis, CSCs preferentially use mitochondrial respiration to obtain sufficient energy for self-renewal [20, 21]. Mitochondrial membrane potential and levels of ROS are reported as elevated in stem-like triple-negative breast cancer cells [22].

Apoptosis induced by mass ROS production is the major method of cell death caused by radiation therapy; therefore, regulators of apoptosis are important factors the response to IR [23]. BCL-2 apoptosis regulator (BCL-2) family proteins play an integral role in apoptosis by governing mitochondrial outer membrane potential, consisting of pro-apoptotic proteins (Bim, Bid, Puma, and Noxa) and antiapoptotic proteins (MCL-1, BCLl-2, and BCL-XL). All family member share a BCL-2 homology (BH) domain; however, MCL-1 is distinguished from the others by its fast turnover [24]. The half-life of MCL-1 may be shortened or lengthened significantly depending on the cellular conditions or environmental stress [25]. In response to IR-induced DNA damage, the balance between pro-apoptotic and antiapoptotic BCL-2 proteins determines cell fate [26–28]. Cancer cells may escape apoptosis by expressing high level of antiapoptotic proteins. The overexpression of antiapoptotic proteins, including BCL-2, BCL-XL and MCL-1, contributes to treatment resistance in stem cell-like cancer cells. Inhibition of BCL-2 family proteins increases the antitumor treatment efficacy [29–32].

In this study, we mimicked the treatment in NPC cells in vitro and developed IR-induced radioresistant cell lines. Then, we investigated the phenotypic and functional characteristics of our models. Furthermore, we reported MCL-1 as a modulator of acquired resistance in NPCs receiving IR treatment.

## Materials and methods

### Cell culture and reagents

Parental (relatively sensitive to IR, has been previously reported [33]) and radioresistant subpopulations of NPC cells were maintained in Dulbecco’s modified Eagle’s medium (DMEM) (Invitrogen, Waltham, MA, USA) supplemented with 10% fetal bovine serum (FBS; Invitrogen) at 37 °C and 5% CO2. Cells were plated in 6-well or 12-well plates (Corning Inc., Corning, NY, USA) and the plating efficiencies of cell lines used are higher than 90%. Cells are treated with cycloheximide (CHX; 66-81-9, Abcam, Cambridge, MA, USA), MG132 (S2619, Selleck, Houston, TX, USA), N-acetylcysteine (S0077, Beyotime, Jiangsu, China), or MK2206 (S1078, Selleck) according to studies.

### Cell clonogenic survival and cell viability assays

Cells (5 × 10^3^) were suspended and seeded into 6-well plates after receiving the indicated dose of irradiation, which utilizing patented x-ray irradiation technology, cultured for 10–12 days, fixed, and stained with 0.4% methylene blue (Sigma-Aldrich, St Louis, MO, USA). Colonies were defined as >50 cells. For the cell viability assay, cells were seeded into a 96-well plate (Corning) and cultured for 4 days. At various time points after seeding, the cells in each well were stained with MTS (3-(4,5-dimethylthiazol-2-yl)−5-(3-carboxymethoxyphenyl)−2-(4-sulfophenyl)−2H-tetrazolium; G5421, Promega, Madison, WI, USA), and the OD490 was determined using a microplate reader.

### Spheroid formation assay

Single-cell suspensions containing 500–800 cells were seeded in 12-well ultra-low-attachment culture plates and cultured in serum-free DMEM/F12 (11320082, Invitrogen) supplemented with 20 ng/mL epidermal growth factor (EGF; PHG0311, Invitrogen) and 10 ng/mL basic fibroblast growth factor (bFGF; PHG0360, Invitrogen) for 10 days. The formed spheroids were counted, and representative images were acquired via microscopy.

### Lentiviral transduction studies

An *MCL1* expression construct was generated by subcloning the PCR amplified, full-length human *MCL1* cDNA into pBABE-puro plasmid; an empty vector (Vec) was used as a control. Cells stably expressing either an *MCL1* short hairpin RNA (shRNA) or a scrambled, nontarget shRNA (shLuc) were established using the LV3 plasmid according to the manufacturer’s instructions [34]. The targets of shRNA-1 (sh1) and shRNA-2 (sh2) were 5′-CTTCCATGTAGAGGACCTAGA-3′, and 5′-GCCTAGTTTATCACCAATAAT-3′, respectively. Virus production and infection were performed as previously described [34], and stable cell lines were selected using 2 mg/mL puromycin for 7 days.

### Flow cytometry

For side population (SP) analysis, the cells were harvested and incubated with Hoechst 33342 dye (5 mg/mL, Sigma) with or without Fumitremorgin C (FTC, ABCG2 inhibitor, 50 μM, Sigma) and incubated in the dark for 90 min at 37 °C with intermittent mixing. The cells were then subjected to flow cytometry.

### ROS analysis

Cells were blocked for 30 min in 5% bovine serum albumin (BSA) and incubated with Dichlorofluorescin-diacetate (DCFH-DA) (S0033S, Beyotime) for 30 min in the dark at room temperature. Images were acquired via a high-throughput confocal microscope (Olympus, Tokyo, Japan).

### Western blotting

Immunoblotting was performed according to the standard method as described previously [34]. Primary antibodies were from Cell Signaling Technology (Danvers, MA, USA), including those recognizing cleaved poly(ADP-Ribose) polymerase (PARP; 5625), caspase-3 (9662), β-actin (4970), glyceraldehyde-3-phosphate dehydrogenase (GAPDH; 2118), α-tubulin (2144), MCL-1 (D35A5), BCL-2 (2872), BCL-xL (2762), octamer-binding protein 4 (OCT4; 2750), Nanog (4903), SRY box 2 (SOX2; 14962), hypoxia inducible factor 1 alpha (HIF-1α; 3716), γH2AX(5438), protein kinase B (AKT) (4685), phospho-AKT (4060), S6 ribosomal protein (2217), phospho-S6 ribosomal protein (4858), eukaryotic translation initiation factor 4E binding protein 1 (4E-BP1; 9644), and phospho-4E-BP1 (9451).

### Real-time reverse transcription-quantitative PCR

The mRNA levels of OCT4, Nanog, SOX2 and MCL-1 were measured by real-time RT-PCR according to the manufacturer’s instructions [35]. Briefly, Total RNA was extracted from cultured cell lines using TRIzol reagent (Invitrogen) and then reverse-transcribed using a cDNA Synthesis Kit (Takara, 6111A). Real-time qPCR was performed using a SYBR PCR Kit (LifeScience, 04707516001). The house keeping gene GAPDH was used as the internal normalization control to calculate the mRNA levels of the different genes.

### Tumor xenograft experiments

The protocol for the xenograft experiments comply with the ARRIVE guidelines, and was approved by the Institutional Animal Care and Use Committee of Sun Yat-Sen University Cancer Center. Female BALB/c nude mice (4 weeks old, 16–18 g; Animal Center of Guangdong Province) were housed in barrier facilities. For the tumorigenicity assay, mice were randomly divided into groups. The indicated number tumor cells (2 × 10^4^, 5 × 10^4^ or 15 × 10^4^) were suspended in 50 μL of culture medium containing 50% Matrigel (356243, BD Biosciences, San Jose, CA, USA) and subcutaneously inoculated into the mice (*n* = 10 per group). The mice were monitored every 3 days to measure tumor formation. All mice were euthanized at 5 weeks after injection. The tumor-initiating cell frequency (TIF) was calculated using extreme limiting dilution analysis (ELDA) software (http://bioinf.wehi.edu.au/software/elda/). For the radiation response in vivo assay, mice were inoculated subcutaneously with S26 and S26-R (radioresistant) cells (5 × 10^6^ in 100 μL of sterile phosphate-buffered saline (PBS), *n* = 6 in each group). MK2206 or vehicle (control) was administered daily by gavage (120 mg/kg). The tumor volume and body weight were recorded every 3 days. At day 32, the mice were euthanized, the primary tumors were weighed, and tumor samples were collected for western blotting analysis.

### Statistics

All statistical analyses were carried out using SPSS 21.0 (IBM Corp., Armonk, NY, USA). The data are presented as the mean ± standard error (SE) of at least three independent experiments. Comparisons between the groups were performed using one-way analysis of variance (ANOVA). A two-tailed Student’s *t*-test was used to compare the data between two groups. All cell culture experiments were performed independently in triplicate at least three times. *p*-values < 0.05 were considered statistically significant.

## Results

### Development of the acquisition of radioresistance in NPC cells after Ionizing radiation

**Fig. 1:**
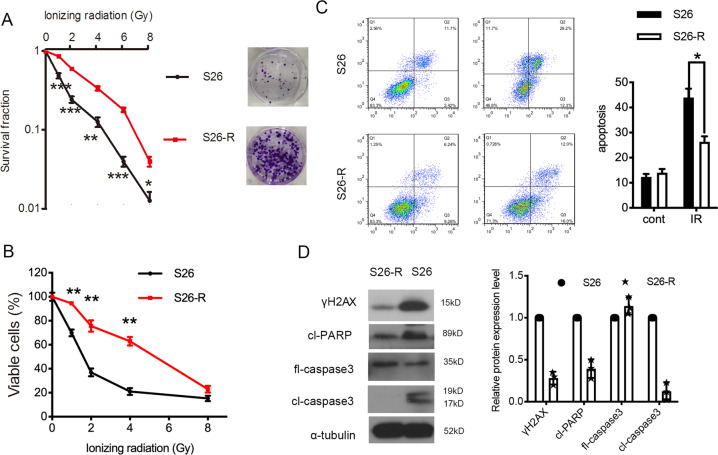
Repeated ionizing radiation induces a risk of radioresistance in NPC cells. **A** (Left) Cell survival curve for treatment with increased doses of ionizing radiation in S26 and S26-R cells, *p < 0.05, **p < 0.01, Student’s *t*-test. (Right) representative images of colonies formed after treatment with the indicated dose of ionizing radiation in S26 and S26-R cells. **B** A cell proliferation curve was constructed from the MTS assay results, data are presented as the mean ± S.D. values, **p < 0.01, Student’s *t*-test. **C**, **D** S26 and S26-R cells were treated with or without 4 Gy of radiation for 48 h, then subjected to flow cytometry analysis of apoptosis *p < 0.05, Student’s *t*-test (**C**) and western blotting of the apoptotic markers (**D**); α-tubulin was used as loading control. (NPC, nasopharyngeal carcinoma; MTS, 3-(4,5-dimethylthiazol-2-yl)−5-(3-carboxymethoxyphenyl)−2-(4-sulfophenyl)−2H-tetrazolium.).

We have reported that S26 cells (a mono-clone derived from CNE-2 cells, a poorly differentiated human NPC cell line) were relatively sensitive to radiation therapy and chemotherapy [33]. To mimic the process of acquired resistance in patients after ionizing radiation (IR) treatment, S26 NPC cells were irradiated by daily exposure (5 days per week, for 6 weeks) to single fractions of 2 Gy X-ray radiation. The acquired radioresistant cell line, S26-R, was developed. Radioresistance was confirmed by growth fraction assays. The survival ability of radioresistant S26-R cells was significantly higher than that of their parental S26 cells when exposed to a gradient dose of radiation (Fig. 1A). Less inhibition of proliferation was also observed in the S26-R cell line under IR (Fig. 1B). To rule out that the effect is cell line specific, we developed radioresistant SUNE1-R cells from another NPC cell line, SUNE1 (Fig. S1A, B). Radiation exerts its cytotoxicity by inducing apoptosis. When exposed to radiation treatment, radioresistant S26-R and SUNE1-R cells showed significantly decreased apoptosis and lower protein levels of H2AX variant histone (γH2AX), cleaved PARP, and cleaved caspase-3, which are all well-known markers of DNA damage and apoptosis, indicating reduced induction of cell death (Fig. 1C, D and Fig. S1C, D).

### Ionizing radiation treatment enriches the CSC subpopulation

**Fig. 2:**
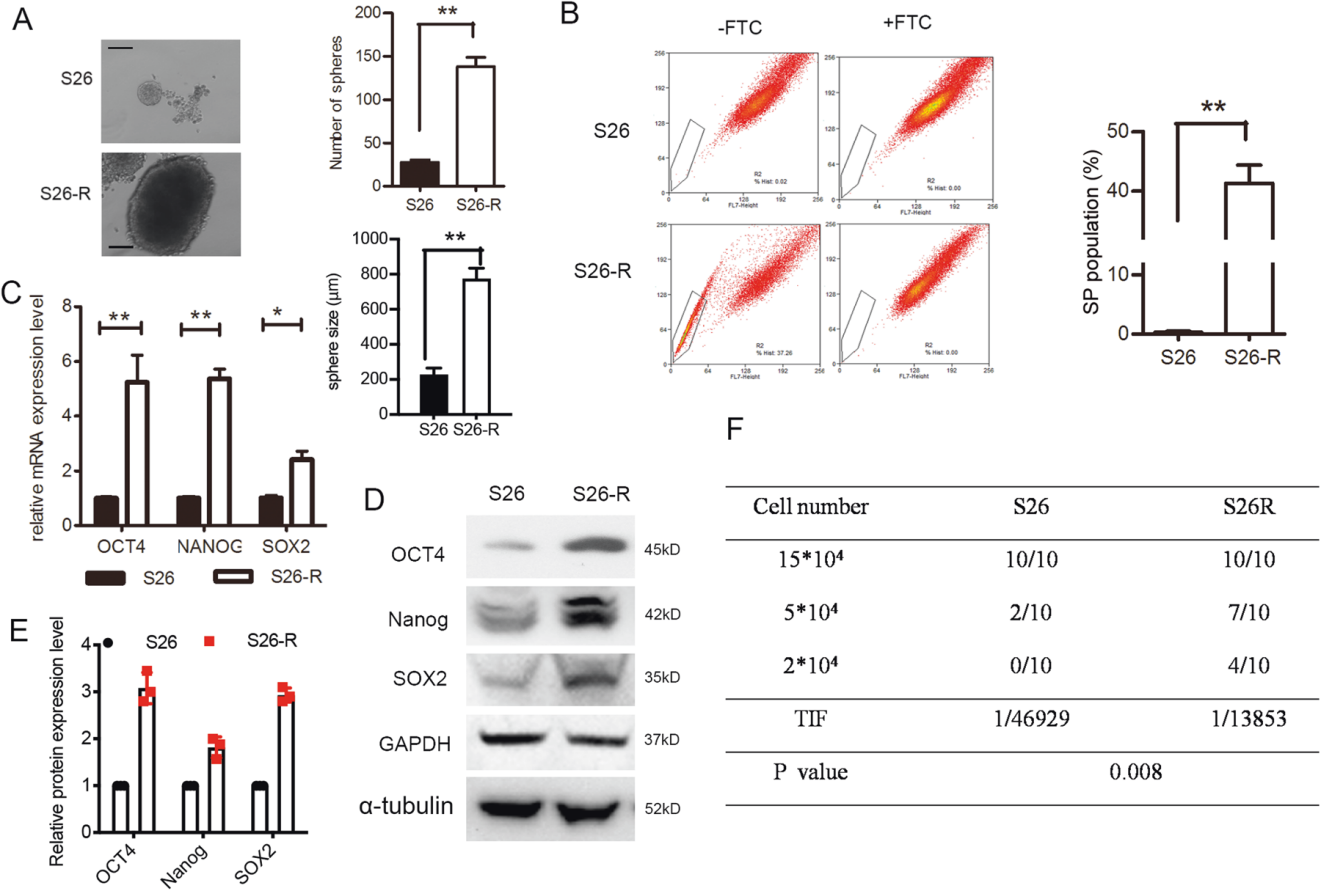
Ionizing radiation enhanced CSC subpopulation enrichment and radioresistance in vitro and in vivo. **A** Single-cell suspensions were seeded in ultra-low-attachment culture plates. The formed spheroids were counted via microscopy. Representative images are shown, the number (upper) and the size (lower) of S26 and S26-R cells were compared, **p < 0.01, Student’s *t*-test, Scale bar: 200 μm. **B** Percentages of SP cells are shown in the left panel; three independent experiments were performed; The right panels show the upregulation of SP in cells S26-R NPC cells, **p < 0.01, Student’s *t*-test. **C** (Left) mRNA levels of stem-cell markers were determined by quantitative PCR analysis, *ACTB* (encoding β-actin) was used as a control. (Right) protein levels of stem-cell markers as determined using western blotting; β-actin was used as the loading control. **D** A total of 2 × 10^4^, 5 × 10^4^, and 15 × 10^4^ of S26 and its radio-resistant S26-R NPC cells were subcutaneously injected into NOD/SCID mice (*n* = 10 mice/group). A summary of tumorigenicity in mice is shown. The TIF and *p*-value were calculated by using ELDA software. (NPC, nasopharyngeal carcinoma; CSC, cancer stem cell; SP, side population; NOD, non-obese diabetic; SCID, severe combined immunodeficiency; TIF, tumor-initiating cell frequency; ELDA, extreme limiting dilution analysis).

Stem cells have the capability to form spheres under suspension culture conditions. The cells that survived after IR showed a significantly increased sphere formation ability and size (Fig. 2A) and the percentage of subpopulation (SP) cells, a CSC marker for NPC cells (*p* < 0.001, Student’s *t*-test; Fig. 2B). Consistently, western blotting and quantitative PCR showed that levels of CSC markers (OCT4, Nanog, and SOX2) were elevated dramatically in radioresistant S26-R and SUNE1-R cells compared with those in their parental cells (Fig. 2C and S2).

Then, we inoculated radioresistant S26-R cells and their parental S26 cells subcutaneously into nude mice at densities of 2 × 10^4^, 5 × 10^4^, and 15 × 10^4^ cells, respectively. Parental S26 cells injected at 15 × 10^4^ cells formed tumors in all nude mice, similar to S26-R cells, but at a much slower rate. When injected at a lower density (5 × 10^4^ cells), 7 of 10 nude mice formed tumors in the S26-R group, while parental S26 cells formed tumors in 2 of 10 nude mice. When 2 × 10^4^ cells were injected, parental S26 cells failed to form tumors in nude mice, whereas tumors formed in four of 10 mice bearing S26-R cells (Fig. 2D and Fig. S3). The above data confirmed that NPC cells after IR showed stem-cell phenotypes.

### The enhanced stem-cell phenotypes induced by IR were MCL-1-dependent

**Fig. 3:**
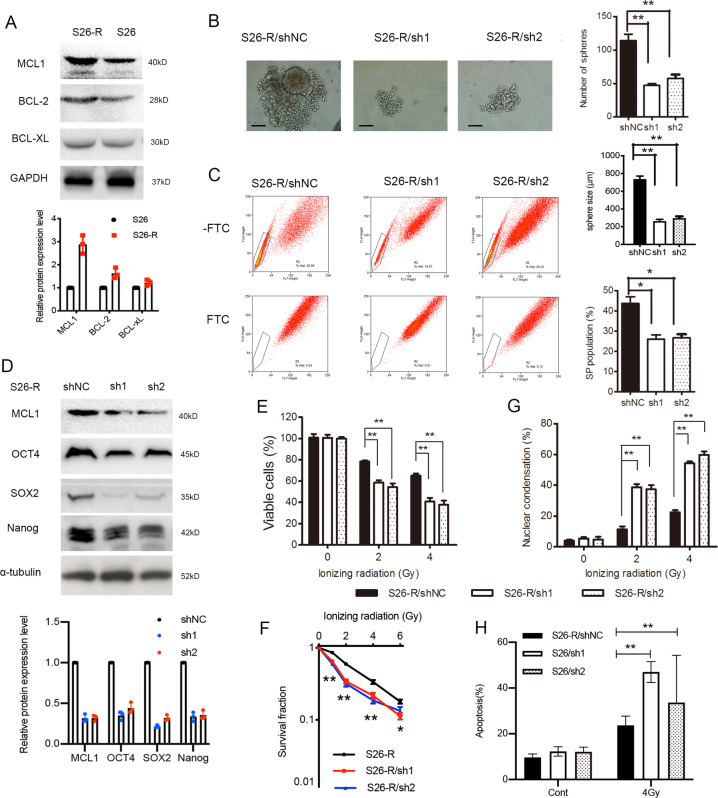
MCL-1 depletion in radioresistant NPC cells inhibited stem-cell characteristic and restored radiation sensitivity. S26 NPC cells were irradiated by daily exposure (5 days per week, for 6 weeks) to single fractions of 2 Gy X-ray radiation to acquire radioresistant cell line S26-R. **A** Western blotting analysis of BCL-2 family members expression in S26 and its radio-resistant S26-R cells. **B**–**F** S26-R cells stably transfected with shRNA targeting *MCL1* (sh1, sh2) or scrambled shRNA (shNC) were analyzed as follows. (**B**, left panel) Representative images of single-cell suspensions in ultra-low-attachment culture plates are shown. (Right panel) The formed spheroids were counted via microscopy (upper) and the size of spheroids were compared (lower), **p < 0.01, Student’s *t*-test, Scale bar: 200 μm. **C** Percentages of SP cells are shown in the left panel, and the right panels compare the SP formed in S26-R cells transfected with the shRNA targeting MCL-1 or its control, *n* = 3; *p < 0.05, Student’s *t*-test. **D** Protein levels of stem-cell markers were determined by western blotting, tubulin was used as the loading control. **E** Cell viability of S26-R cells transfected with shRNA targeting MCL-1 or its control after treatment with ionizing radiation determined by the MTS assay, **p < 0.01, Student’s *t*-test. **F** Cell survival curve for treatment with increased doses of ionizing radiation in S26-R cells transfected with shRNA targeting MCL-1 or its control, *p < 0.05, **p < 0.01, Student’s *t*-test. **G**, **H** S26-R cells transfected with shRNA targeting *MCL1* or its control were treated with indicated dose of radiation, then necrotic cells are revealed by propidium iodide staining (**G**) and apoptotic cells are revealed by flow cytometry analysis (**H**). Qualification results are shown, **p < 0.01, Student’s *t*-test. (NPC, nasopharyngeal carcinoma; MTS, 3-(4,5-dimethylthiazol-2-yl)-5-(3-carboxymethoxyphenyl)-2-(4-sulfophenyl)-2H-tetrazolium; shRNA, short hairpin RNA; SP, side population).

Increased antiapoptotic BCL-2 protein expression has been implicated in the development of resistance to radiation therapies. In our models, higher levels of MCL-1 and BCL-2 were found in S26-R and SUNE1-R cells after exposure to radiation, while nonsignificant changes in BCL-xL were also observed (Fig. 3A and Fig. S4A).

To test the role of MCL-1 in NPC CSC subpopulation enrichment, we knocked down *MCL1* and found that its depletion reduced the sphere formation ability/size of radioresistant NPC cells (Fig. 3B). Knockdown of *MCL1* reduced the percentage of SP cells compared with that in the parental S26 cells (Fig. 3C, from 43.6% to 25.7% and 26.6%, respectively, *p* < 0.05, Student’s *t*-test) and decreased the levels of CSC markers, as indicated by western blotting analysis (Fig. 3D). Moreover, proliferation assays showed that silencing *MCL1* expression contributed to inhibition of cell proliferation (Fig. 3E) and clonogenic cell survival (Fig. 3F). Ionizing radiation-induced DNA damage results in an increase in nuclear chromatin condensation [36]. Knockdown of *MCL1* increased nuclear condensation and nuclear fragmentation significantly when exposed to a gradient dose of radiation (Fig. 3G and Fig. S5A; *p* < 0.01 for both comparisons). Radiation induced significantly increased apoptosis in cells with *MCL1* silencing (Fig. 3H). Additionally, we observed a significant decreased in sphere formation and vulnerability to IR treatment in SUNE1-R cells when *MCL1* was knocked down (Fig. S4B–D).

**Fig. 4:**
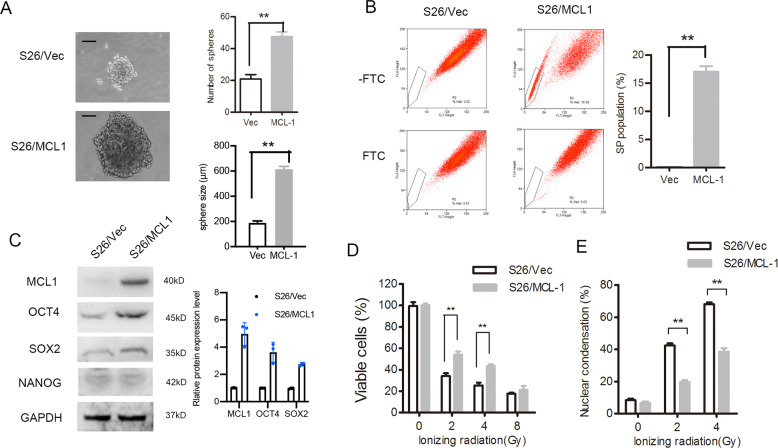
Expression of MCL-1 in NPC cells induced CSC characteristic and radiation resistance. S26 cells stably overexpressing MCL-1 or empty vector (Vec) were analyzed as follows. **A** The representative images of single-cell suspensions are shown (left panel) and spheroids were counted (right upper panel), and the size were measured (right lower panel), **p < 0.01, Student’s *t*-test. **B** Percentages of SP cells are shown in the left panel, and the right panels compare the populations of SP, *n* = 3, *p < 0.05, Student’s *t*-test. **C** Protein levels of stem-cell markers were determined by western blotting, GAPDH was used as the loading control. **D** Cell viability after treatment with ionizing radiation determined by MTS assay, **p < 0.01, Student’s *t*-test. **E** Qualification of nuclear condensation after treatment with different doses of radiation are shown, **p < 0.01, Student’s *t*-test. (NPC, nasopharyngeal carcinoma; CSC, cancer stem cell; MTS, 3-(4,5-dimethylthiazol-2-yl)-5-(3-carboxymethoxyphenyl)-2-(4-sulfophenyl)-2H-tetrazolium; SP, side population).

Consistently, the overexpression of *MCL1* increased the sphere formation ability and the size of S26 cells (Fig. 4A). S26 cells with high levels of MCL-1 increased the percentage of the SP cells (Fig. 4B, from 0.07% to 16.8%, *p* < 0.001, Student’s *t*-test) and stem-cell marker expression (Fig. 4C). Moreover, increasing MCL-1 levels contributed to radioresistance (Fig. 4D) and decreased nuclear condensation (Fig. 4E and Fig. S5B) upon exposure to radiation.

### Activation of the ROS-AKT feedback loop regulates the protein synthesis of MCL-1 in vitro

**Fig. 5:**
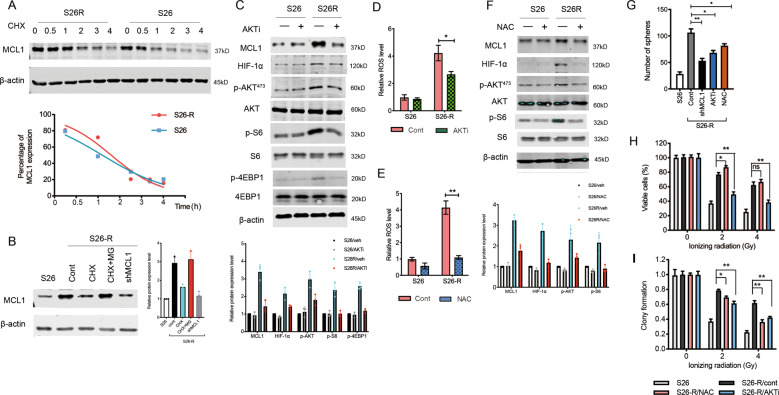
ROS-AKT feedback loop activation regulates the protein synthesis of MCL-1. **A** S26 and S26-R cells were treated with CHX (50 μg/mL), lysates were collected at the indicated times. (Upper) Immunoblotting analysis of MCL-1 levels. (Lower) MCL-1 levels were quantified and normalized to the signal of β-actin. **B** S26-R and S26 cells were treated with CHX (50 μg/mL) or combined with MG132 (5 μM) for 2 h. Cell lysates were subjected to western blotting. β-actin was used as the loading control. **C**, **D** S26-R and S26 cells were treated with the AKT inhibitor MK2206 (1 μM) for 2 h, and then cell lysates were subjected to immunoblotting (**C**), β-actin was used as the loading control, and quantification of ROS levels is shown (**D**) (*n* = 3); *p < 0.05, **p < 0.01 compared with the 0 h control. **E**, **F** S26 and S26-R cells were treated with N-acetylcysteine (NAC, 5 μM) for 2 h, **E** Quantification of ROS levels measured by DCFH-DA is shown (*n* = 3; *p < 0.05, **p < 0.01 compared with the 0 h control); **F** cell lysates were subjected to immunoblotting, β-actin was used as the loading control. **G** S26-R cells and s26 cells were treated with the AKT inhibitor or NAC, then the cells were cultured for 10 days, and the formed spheroids were counted and compared, **p < 0.01, *p < 0.05, Student’s *t*-test. **H**, **I** S26-R cells and S26 cells were treated with the AKT inhibitor or NAC for 2 h, and then cells received the indicated dose of irradiation, and cell viability was determined by the MTS assay 2 days post-irradiation, and clony formation was determined by stained with methylene blue and counted 10 days post-irradiation, **p < 0.01, *p < 0.05, Student’s *t*-test. (CHX, cycloheximide; ROS, reactive oxygen species; DCFH-DA, dichlorofluorescin-diacetate; MTS, 3-(4,5-dimethylthiazol-2-yl)-5-(3-carboxymethoxyphenyl)-2-(4-sulfophenyl)-2H-tetrazolium).

Regulation of MCL-1 expression occurs at the transcriptional, translational, and posttranslational levels. We measured the mRNA levels of *MCL1* using quantitative PCR and found that the mRNA level was not altered in radioresistant S26 and SUNE1 NPC cells (Fig. S6A). We treated cells with CHX, a translation inhibitor, and collected protein at indicated time points to test existing MCL-1 protein levels. The results showed that MCL-1 protein stability was increased slightly in S26-R cells when compared with that in S26 cells (Fig. 5A). Then, the MCL-1 synthesis level was compared between S26 and S26-R cells treated with cycloheximide to remove MCL-1 proteins and then treated with MG132 to obtain newly synthesized proteins. Radioresistant S26-R cells exhibited increased synthesis of the MCL-1 protein (Fig. 5B).

The process of protein synthesis is active during mitosis. DNA content analysis by fluorescence microscopy confirmed that S26-R cells had arrested at the G2/M phase, and showed polyploid multinucleate cell formation (Fig. S6B), suggesting that S26-R cells had advantage for protein synthesis. The AKT-mechanistic target of rapamycin (mTOR) signaling pathway has been reported to be involved in the protein synthesis process [37, 38]. To further investigate the mechanism of increased synthesis of MCL-1 in IR-induced NPC cells, we tested the AKT pathway signal and found it was activated in S26-R cells. The AKT inhibitor MK2206 efficiently blocked the activation of AKT, as detected by a decrease in AKT and its downstream ribosomal S6 kinase or 4EBP1 phosphorylation, which consequently caused downregulation of MCL-1 protein expression (Fig. 5C).

High levels of ROS are the main driver for cell death induced by irradiation. However, persistent ROS could be a key step in the transformation of cancer cells to CSCs [39, 40]. To determine the effects of ROS generation between IR-treated and untreated NPC cells, we performed an DCFH-DA assay. We observed an obvious increase in ROS soon after radiation and a quick decrease after 8 h, and then a slow decrease to the basal level over 1 to 2 weeks in S26 cells (Fig. S7A). S26-R cells have higher basal level of ROS without treatment when compared with S26 (fold- change = 1.95 ± 0.25, Fig. S7B). However, S26-R cells displayed relatively lower level of ROS generation in the first day after IR treatment (fold-change = 0.86 ± 0.05 in 0 h; fold-change = 0.68 ± 0.11 in 8 h, Fig. S7B), but a significant increase after 2 days(fold-change = 3.45 ± 0.36, Fig. S7B) through to 1 week when compared with S26 cells, suggesting a persistently higher basal level of ROS in the process of CSC formation (Fig. S7B). AKT inhibitor treatment cause a slight decrease in HIF-1α levels (a direct target of ROS) and significant downregulation of intracellular ROS levels (Fig. 5C, D). Then, N-acetylcysteine (NAC) was applied to inhibit basal ROS levels, and the efficiency of NAC was confirmed by reduced ROS and HIF-1α levels. Downregulation of AKT and phosphorylation of its downstream ribosomal S6 kinase were consistently observed (Fig. 5E, F). To confirm the impact of ROS-AKT on MCL1 synthesis, we use another NPC cell line, SUNE1, to test MCL1 level under inhibitor and NAC treatment. Again, we observed AKT inhibitor treatment lead to MCL1, HIF-1α downregulation and ROS level (Fig. S8A, B). N-acetylcysteine was also applied to treat SUNE1 cells. The effect of NAC was confirmed by reduced ROS level and HIF-1α expression (Fig. S8C, D). Interestingly, NAC treatment benefited MCL1 inhibition (Fig. S8C).

The above data suggested that the ROS-AKT axis forms a positive feedback loop that regulates MCL-1 protein synthesis, which is closely related to the CSC phenotype induced by irradiation (Fig. 5G, H, I and S8E). Inhibition of AKT increased the sensitivity of radioresistant cells to radiation therapy. Unexpectedly, ROS inhibition failed to reversed resistance to IR (Fig. 5H). We suspect that elevated intracellular ROS causes lethal damage induced by irradiation, allowing ROS inhibition to improve cell survival while receiving irradiation.

### Inhibition of AKT-MCL-1 signaling increased the antitumor effect of radiation therapy in vivo

**Fig. 6:**
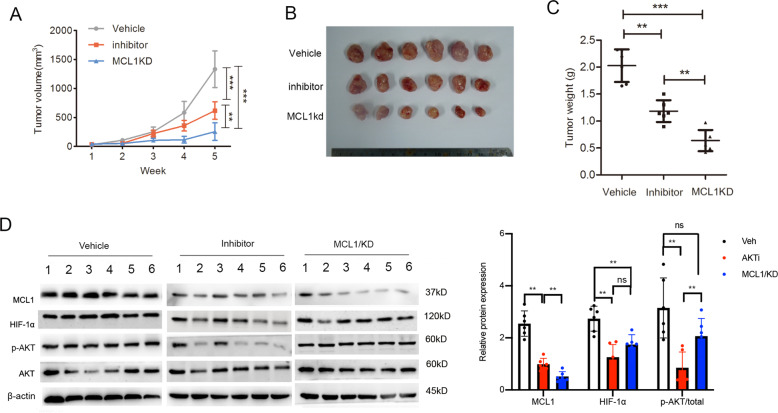
Inhibition of AKT-MCL-1 signaling increased antitumor effect of radiation therapy in vivo. S26-R cells transfected with MCL-1 shRNA(MCL1/kd) or negative control shRNA were injected subcutaneously into BALB/c nude mice. Then, S26-R cells transfected with the control scrambled shRNA were treated with AKT inhibitor (MK2206, AKTi) or vehicle (veh, used as control) during treatment with radiation. **A** Tumor volume growth curves; data are shown as the mean ± SD. (**p < 0.01, ***p < 0.001, Student’s *t*-test). **B** Images of tumors from all mice in the indicated groups (*n* = 6/group). **C** Weight of tumors formed in the indicated groups; data are shown as the mean ± SD. (**p < 0.01, ***p < 0.001, Student’s *t*-test). **D** Tumor lysates were subjected to immunoblotting, β-actin was used as the loading control (left panel). Levels of the indicated proteins were quantified and normalized to the signal of β-actin, and phospho-AKT were normalized total AKT and β-actin (right panel).

As described above, the AKT inhibitor acted as a tool to reverse radioresistance. To examine the effect of AKT-MCL-1 signaling on radiation sensitivity in vivo, nude mice were injected subcutaneously with S26-R cells transfected with *MCL1* shRNA1 or control shRNA. All mice received daily therapy. Mice injected with control S26-R cells were randomly assigned to receive the AKT inhibitor or vehicle (control) treatment. The weights of the mice in each group were comparable (data not shown), suggesting that the treatment was tolerable for the mice. Tumor-bearing S26-R cells with MCL-1 depletion grew slowly compared with S26-R cells with control shRNA treatment. Interestingly, the AKT inhibitor treatment inhibited the growth of the tumors formed by S26-R cells, but not to the same extent as that of MCL-1 depletion (Fig. 6A–C). Immunoblotting of tumor lysates showed an obvious reduction in MCL-1 levels in the MCL-1 knockdown group, but little effect on AKT/ HIF-1α pathway molecules while compared with vehicle treatment group, suggesting that MCL-1 acts as a downstream factor in the AKT/ HIF-1α signaling pathway. AKT inhibitor treatment reduced MCL-1 protein levels, even though the reduction was not comparable to MCL-1 knockdown in the tumors. We also observed the size of tumor in mice was closely correlated with MCL-1 expression. Moreover, AKT inhibitor significantly suppressed HIF-1α and phosphor-AKT expression, indicating HIF-1α, at least partially, was regulated by AKT pathway (Fig. 6D).

## Discussion

MCL-1-mediated chemotherapy resistance was attributed to resistance to apoptotic cell death or compromised homologous recombination-mediated DNA double-strand break repair [41]. Furthermore, the correlation between MCL-1 overexpression and stem cell-like potential has been reported previously in several cancer types, such as breast cancer [42], leukemia [43, 44], lung cancer [45], and glioblastoma [46]. In our study, we examined an additional role for MCL-1 in irradiation-induced cancer stem-like cells and acquired radioresistance in NPC.

Regulation of MCL-1 expression occurs at the transcriptional, translational, and posttranslational levels. In response to stimuli, such as growth factors or cytotoxic drugs, *MCL1* transcription is enhanced by activating transcription factors (ATF5, STAT, and HIF-1α) [47, 48]. Despite the suggestion that HIF-1α activates *MCL1* transcription, we found that the *MCL1* mRNA level remained relatively constant even though HIF-1α expression was increased significantly. This observation ruled out transcriptional regulation as being responsible for IR-induced MCL-1 upregulation.

MCL-1 differs from other BCL-2 family members by a unique characteristic: It can be modified on sites within PEST (proline, glutamic acid, serine, and threonine) on its N-terminal region, affecting its rate of turnover [27, 49]. The degradation of MCL-1 was mainly attributed to the presence of amino acid residues marked for ubiquitination by USP9X when exposed to irradiation, which further promoted radioresistance [50]. Other posttranslational modifications (phosphorylation, caspase cleavage) are also dispensable for MCL-1 stability, closely correlating with the response to targeted therapy, chemotherapeutics, and oxidative stress [51–57]. Consistent with previous studies, MCL-1 downregulation was slightly delayed after proteasome inhibitor treatment in IR-induced radioresistant NPC cells during the observation period. Moreover, we observed an increase in MCL-1 synthesis in our model. MCL-1 protein synthesis has been reported to be blocked in Hela cells following a single fraction of ultraviolet irradiation [58]. Early-phase repression of protein synthesis, which helps to save energy in times of cellular stress, would not last long, with recovery of protein synthesis delayed until 48 h following high-dose IR treatment [59]. However, in cells that survived under multiple fractionated irradiation, enhanced protein synthesis later becomes the predominant mode [60]. Thus, our results suggested that MCL-1 accumulation is caused by an increase in MCL-1 stability and more importantly, enhanced protein synthesis.

Changes in protein synthesis capacity are required to maintain the demands of proliferating tumor cells [61]. Multiple mechanisms appear to participate in the process of protein synthesis. Upregulated phosphorylation of eIF4A, a key component of the translation and downstream effector of the AKT signaling pathway, plays a vital role [61]. AKT inhibition downregulated the phosphorylation of 4EBP1 and S6 and markedly reduced the levels of MCL-1 [62]. The AKT/ MCL-1 signaling pathway has been reported to play a prominent role in mediating antiapoptotic signals in chronic lymphocytic leukemia B cells [63] or in resistance to BCL-2/PARP inhibitors, or BH3 mimetics [64–66]. Enhanced AKT/mTOR signaling also contributes to X-ray and carbon ion beam irradiation resistance [67]. Our results demonstrated that the AKT signaling pathway controls the protein synthesis of MCL-1 in vitro (Fig. 6). Targeting AKT achieved limited success compared with *MCL1* knockdown, and we suspected that the reason for the disappointing activity of the drugs was the induction of multiple feedback loops that causes overactivation of upstream or compensatory pathways, including PI3K and ERK, potentially blocking the antitumor effects of the inhibitors [68, 69]. A combination of bypass inhibitors could be applied to attenuate reactivation of the AKT signaling pathway.

Anti-cancer strategies always include chemotherapeutic drugs or irradiation to mediating ROS enhancement. High concentration of ROS induced by ionizing radiation is a key factor in the damage effect of the body. Reducing ROS with NAC treatment benefited cell survival at short-term post-irradiation (Fig. 5H and S8E). However, inhibition of ROS production significant hinder cell colony formation in long term (Fig. 5I). In fact, growing evidence supports the view that a moderate level of ROS is required for some cellular functions, including cell proliferation and angiogenesis, which are vital for tumorigenesis [70]. Cancer cells normally adapt to persistent oxidative stress by enriching their stem-like potential [71]. Human breast CSCs maintain persistent ROS generation that provide radioprotection, representing a possible explanation for tumor recurrence with therapy [18, 72]. Persistent prolonged ROS-induced oxidative stress expands the clonal selection of cancer cells, gradually making these clonal cells form subsets with new features that surviving from radiation exposure [73].

Small increases in ROS would be expected to activate the PI3K/AKT pathway [74–77]. In contrast, PI3K/AKT signaling contributes to increased ROS levels through direct modulation of mitochondrial bioenergetics, or indirectly through the production of ROS as a metabolic by-product [78]. In the present study, we found inhibition of AKT pathway helps reduction of ROS generation in vivo and in vitro. We also showed that ionizing radiation produced large amounts of ROS rapidly, which then decreased at 8 h post-irradiation, and maintained a consistently higher basal level subsequently, suggesting a relatively increased basal level of ROS is involved in the IR-induced stem-cell phenotype. Inhibition of ROS, however, failed to reverse cellular resistance to irradiation (Fig. 5G), strongly indicating the indispensable role of high concentration of ROS in IR-induced cell death. The synthesis of MCL-1 is controlled by a positive feedback loop of ROS/AKT signaling, which helps to maintain persistent intracellular MCL-1 levels. Although chronic oxidative stress is critical to the CSC phenotype induced by IR, redox cancer drugs have not yet showed convincing antitumor activity. How to maintain the subtle balance of intracellular ROS levels to increase treatment success remains a significant challenge.

In summary, we have shown that ionization radiation induces positive feedback activation of the ROS/AKT axis, which mediates increased synthesis of MCL-1, and contributes to CSC enrichment. In turn, CSC enrichment leads to resistance to radiation therapy in irradiated NPC tumors. Thus, a combinatorial approach of depleting MCL-1 in conjunction with IR might provide an important therapeutic improvement for the IR-induced resistant NPC.

## Supplementary information

revided supplementary description-without redmarks

Figure.S1

Figure.S2

Figure.S3

Figure.S4

Figure.S5

Figure.S6

Figure.S7

Figure.S8

CHECKLIST

agreement of co-authors

author contribution

conflict of interest

## Data Availability

The data used to support the findings of this study are included within the article and supplementary. Otherwise, some data are available from the corresponding author on reasonable request.
