# Supplementary material for: Increased MCL-1 synthesis promotes irradiation-induced nasopharyngeal carcinoma radioresistance via regulation of the ROS/AKT loop
Source: Cell Death Dis. 2022 Feb 8;13(2):131. doi: 10.1038/s41419-022-04551-z (PMC8827103; doi:10.1038/s41419-022-04551-z)
Supplement: Supplementary file 1 — revided supplementary description-without redmarks [file 41419_2022_4551_MOESM1_ESM.docx]

**Fig. S1 Ionizing radiation induces radio-resistance of SUNE1 cells**

(A) (left) Cell survival curve with treatment of increased dose of ionizing radiation in SUNE1 and SUNE1-R cells, * p < 0.05, **p < 0.01, Student’s t-test. (right) Representative images of colonies formed after treatment with ionizing radiation. (B) A cell proliferation curve was constructed from MTS assay results, the data are presented as the mean ± SD values, **p < 0.01, Student’s t-test. (C, D) SUNE1 and SUNE1-R cells were treated with or without 4Gy of radiation for 48 hours, then subjected to flow cytometry analysis of apoptosis *p < 0.05, Student’s t-test(C) and western blotting of the apoptotic markers(D); α-tubulin was used as the loading control.

**Fig. S2 Radiation-induced resistance enriches the CSC subpopulation of SUNE1 NPC cells**

(A) Single cell suspensions were seeded in ultra-low-attachment culture plates. The formed spheroids were counted via microscopy, and representative images are shown and the number of SUNE1 and SUNE1-R cells were compared, ***p <* 0.01, Student’s t-test. (B) Protein levels of stem cell markers were determined by western blotting; β-actin was used as the loading control. (NPC, nasopharyngeal carcinoma; CSC, cancer stem cell.)

**Fig. S3 Radiation enriches the CSC subpopulation of NPC cells *in vivo***

(Upper) Tumors formed in mice implanted with indicated number of S26 and its radio-resistant S26-R cells, Scale bar: 1 cm, blue arrow: Tumor formed by S26 cells; red arrow: Tumor formed by S26-R cells. (Bottom) Percentage of tumors formed in mice are compared between S26 and its radio-resistant S26-R cells. (NPC, nasopharyngeal carcinoma; CSC, cancer stem cell.)

**Fig. S4 Knockdown of *MCL1* reduces the CSC subpopulation of radioresistant SUNE1 NPC cells**

SUNE1 NPC cells were irradiated by daily exposure (5 days per week, for 2 months) to single fractions of 2 Gy X-ray radiation to acquire radioresistant cell line SUNE1-R. (A) The lysate of SUNE1 cells and their radio-resistant SUNE1-R cells were subjected to immunoblotting; β-actin was used as the loading control. (B, C, D) SUNE1-R cells stably transfected with shRNAs targeting *MCL1* (sh1, sh2) or scrambled shRNA (shNC) were confirmed with western blotting analysis(B); (C)Single‑cell suspensions were seeded in ultra-low-attachment culture plates, and the formed spheroids were compared, ***p* < 0.01, Student’s t-test. (D) Cell viability after treatment with ionizing radiation determined by the MTS assay, ***p*<0.01, Student’s t-test. (MTS, 3-(4,5-dimethylthiazol-2-yl)-5-(3-carboxymethoxyphenyl)-2-(4-sulfophenyl)-2H-tetrazolium; NPC, nasopharyngeal carcinoma; CSC, cancer stem cell; shRNA, short hairpin RNA.)

**Fig. S5 Representative images of PI staining**

(A) S26-R cells transfected with shRNA targeting *MCL1* or its control were treated with the indicated dose of radiation and necrotic cells are revealed by propidium iodide (PI) staining. Representative images of PI stained cells are shown. Scale bar: 200 μm; arrow: Nuclear condensation.­­ (B) S26 cells stably overexpressing MCL-1 or empty vector (Vec) were treated with indicated dose of radiation and necrotic cells were revealed using PI staining. Representative images of PI-stained cells are shown. Scale bar: 200 μm; arrow: nuclear condensation.­­ (shRNA, short hairpin RNA)

**Fig. S6 MCL1 mRNA expression and cell cycle analysis of radiation sensitive and resistant NPC cells**

(A) mRNA expression level of MCL1 by qPCR analysis in sensitive and its radio-resistant NPC cells, ns, non sinificant. (B)Histograms of the DNA content of singlet cells are shown. Adherent cells were collected, fixed, and stained with propidium iodide (PI), and then examined by fluorescence flow cytometry. Fractions of cells with 2N, 4N, and 8N DNA content represents diploid, tetraploid, and octoploid genomes. (NPC, nasopharyngeal carcinoma.)

**Fig. S7 Representative images of ROS generation**

S26 and S26-R cells were treated with 4Gy of radiation and then stained with CM-H_2_DCFDA to test the intracellular level of ROS. (A) Fluorescence intensity of CM-H_2_DCFDA staining was quantified for unirradiated(cont), and 0 h, 8 h (0.33 day), 2 d, 4 d, 7 ds after ionizing radiation, the results are represented as mean ± SD, n = 3. (B) ROS levels measured by CM-H_2_DCFDA in S26 and S26-R cells at the basal level or after treatment with ionizing radiation. (CM-H_2_DCFDA, chloromethyl derivative of H_2_-Dichlorofluorescin-diacetate; ROS, reactive oxygen species.)

**Fig. S8 ROS-AKT feedback loop has an impact on MCL1 synthesis of SUNE1 NPC cells**

(A, B) SUNE1-R and SUNE1 cells were treated with the AKT inhibitor MK2206 (1 μM) for 2 h, and then cell lysates were subjected to immunoblotting, protein levels were quantified and normalized to the signal of β-actin(A), and quantification of ROS levels is shown (B) (n = 3); **p* < 0.05, compared with the 0 h control. (C, D) SUNE1-R and SUNE1 cells were treated with N-acetylcysteine (NAC, 5 μM) for 2 h, and then cell lysates were subjected to immunoblotting, protein levels were quantified and normalized to the signal of β-actin(C), and quantification of ROS levels measured by DCFH-DA is shown(D) (n = 3; ***p*< 0.01 compared with the 0 h control); (E) SUNE1-R and SUNE1 cells were treated with the AKT inhibitor or NAC for 2 h, and then cells received the indicated dose of irradiation, and cell viability was determined by the MTS assay, ***p* < 0.01, Student’s t-test
