# Supplementary figures and images for: Increased MCL-1 synthesis promotes irradiation-induced nasopharyngeal carcinoma radioresistance via regulation of the ROS/AKT loop
Source: Cell Death Dis. 2022 Feb 8;13(2):131. doi: 10.1038/s41419-022-04551-z (PMC8827103; doi:10.1038/s41419-022-04551-z)

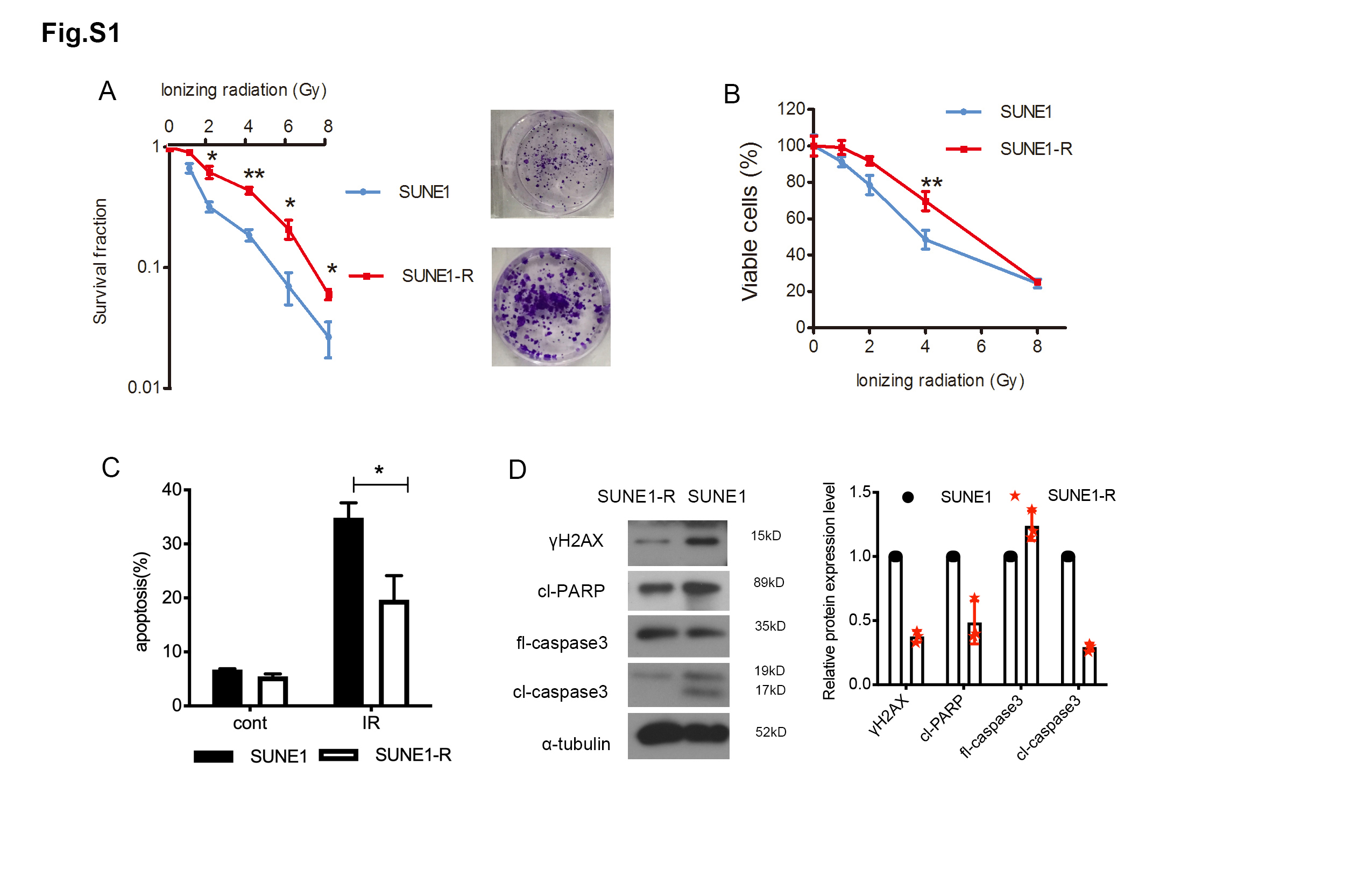

Supplement: Supplementary file 2 — Figure.S1 [file 41419_2022_4551_MOESM2_ESM.jpg]

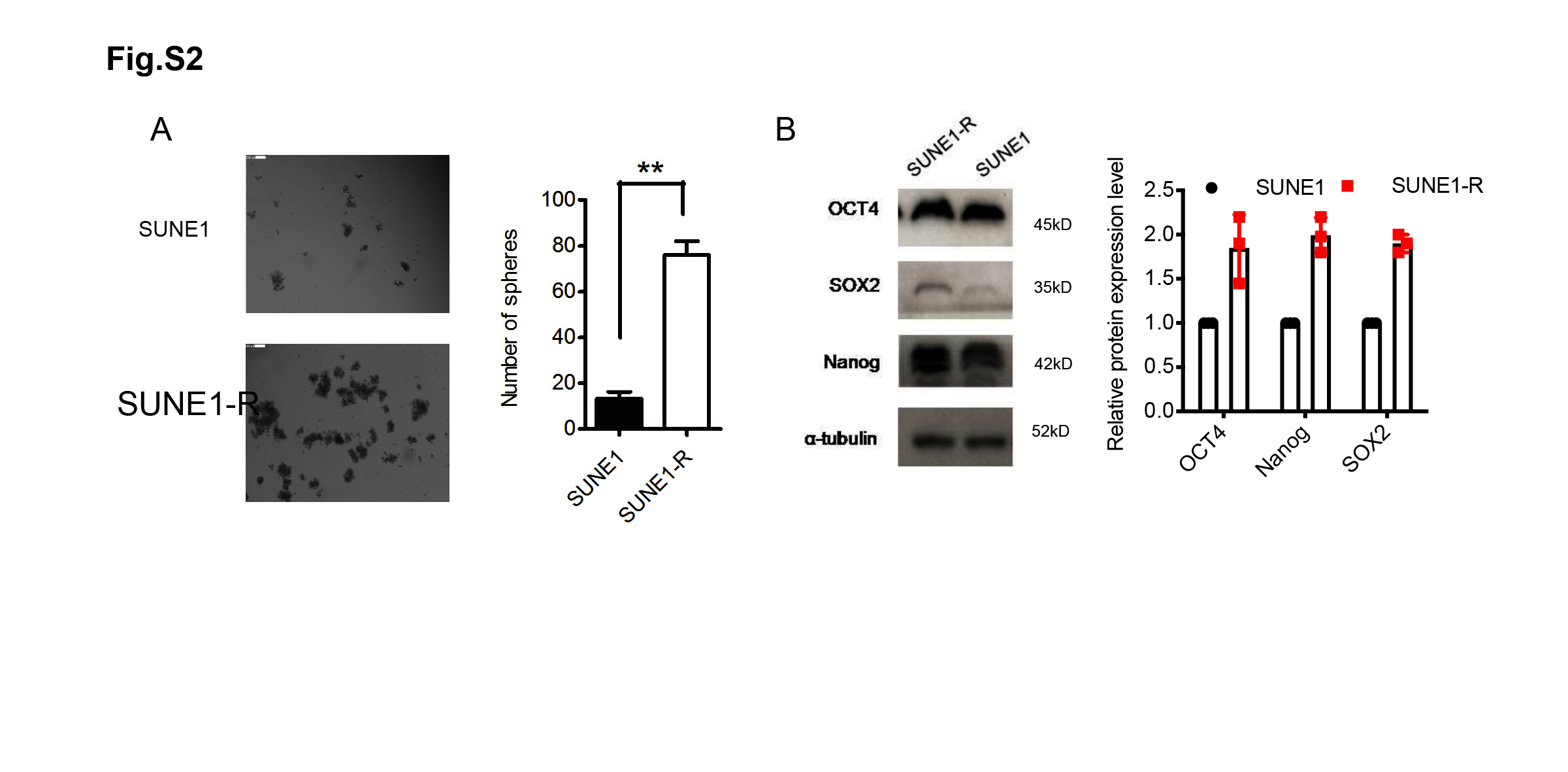

Supplement: Supplementary file 3 — Figure.S2 [file 41419_2022_4551_MOESM3_ESM.jpg]

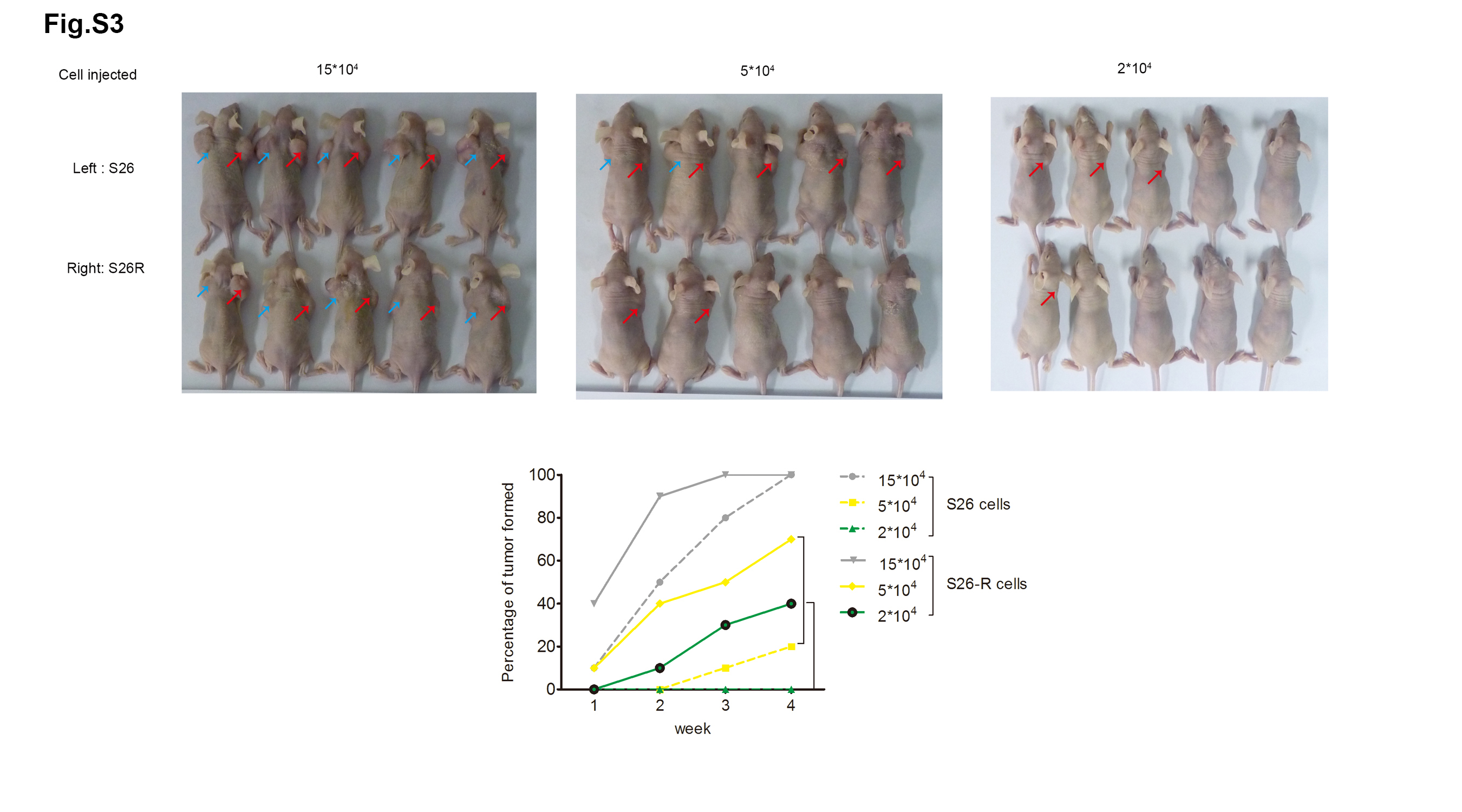

Supplement: Supplementary file 4 — Figure.S3 [file 41419_2022_4551_MOESM4_ESM.jpg]

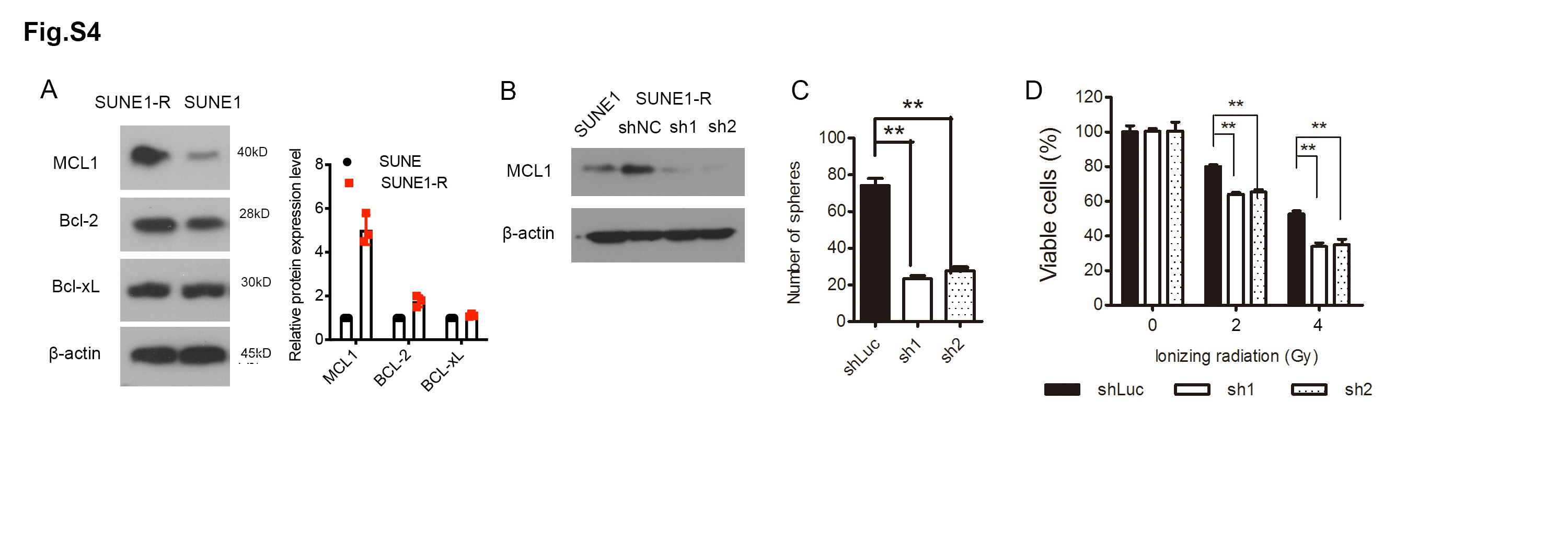

Supplement: Supplementary file 5 — Figure.S4 [file 41419_2022_4551_MOESM5_ESM.jpg]

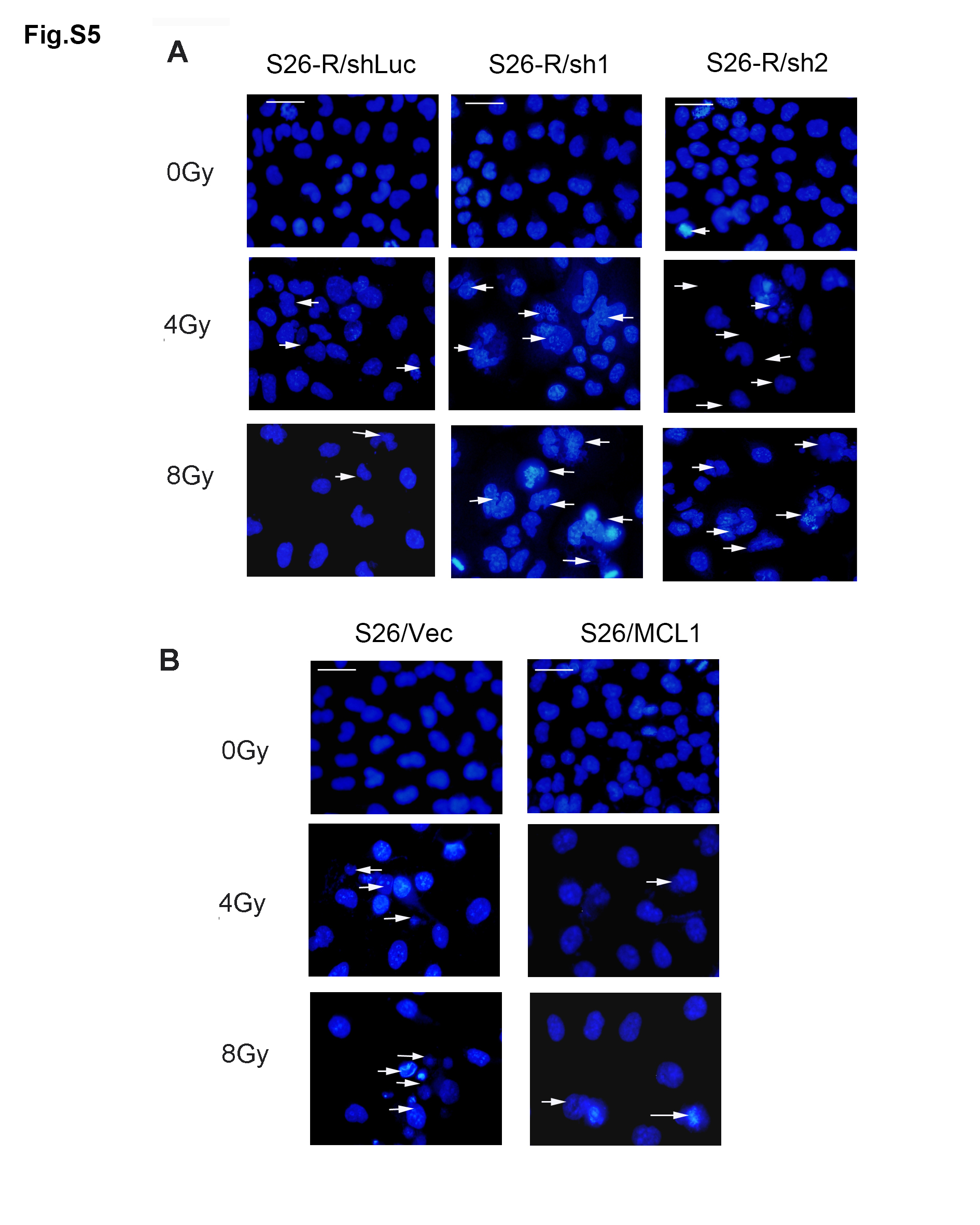

Supplement: Supplementary file 6 — Figure.S5 [file 41419_2022_4551_MOESM6_ESM.jpg]

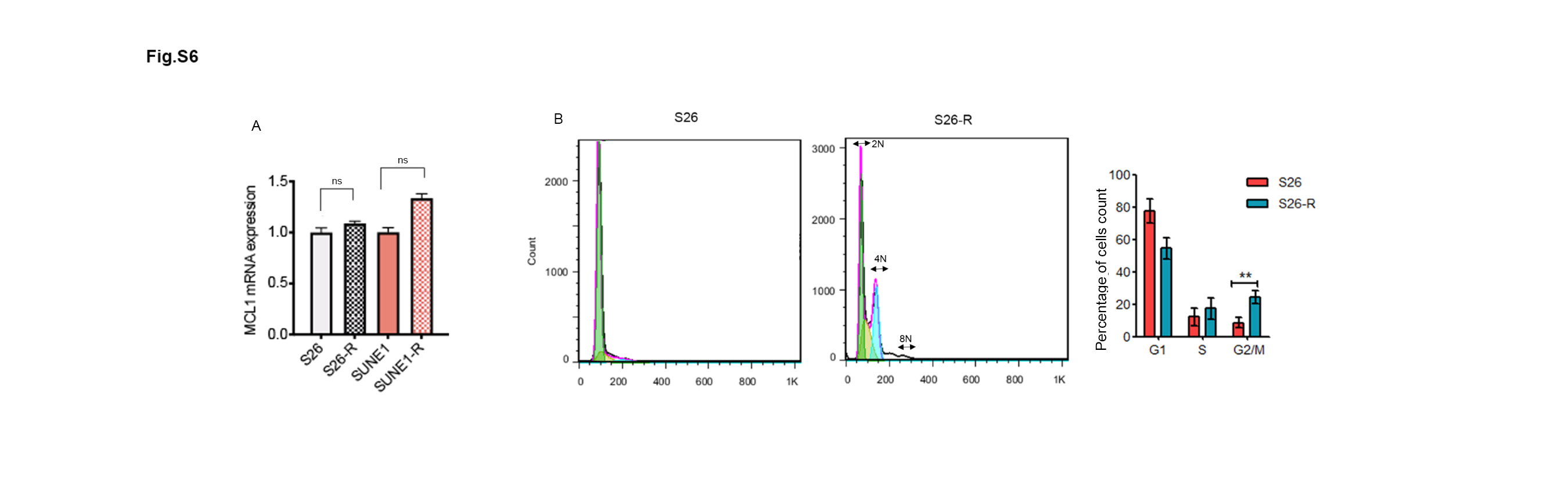

Supplement: Supplementary file 7 — Figure.S6 [file 41419_2022_4551_MOESM7_ESM.jpg]

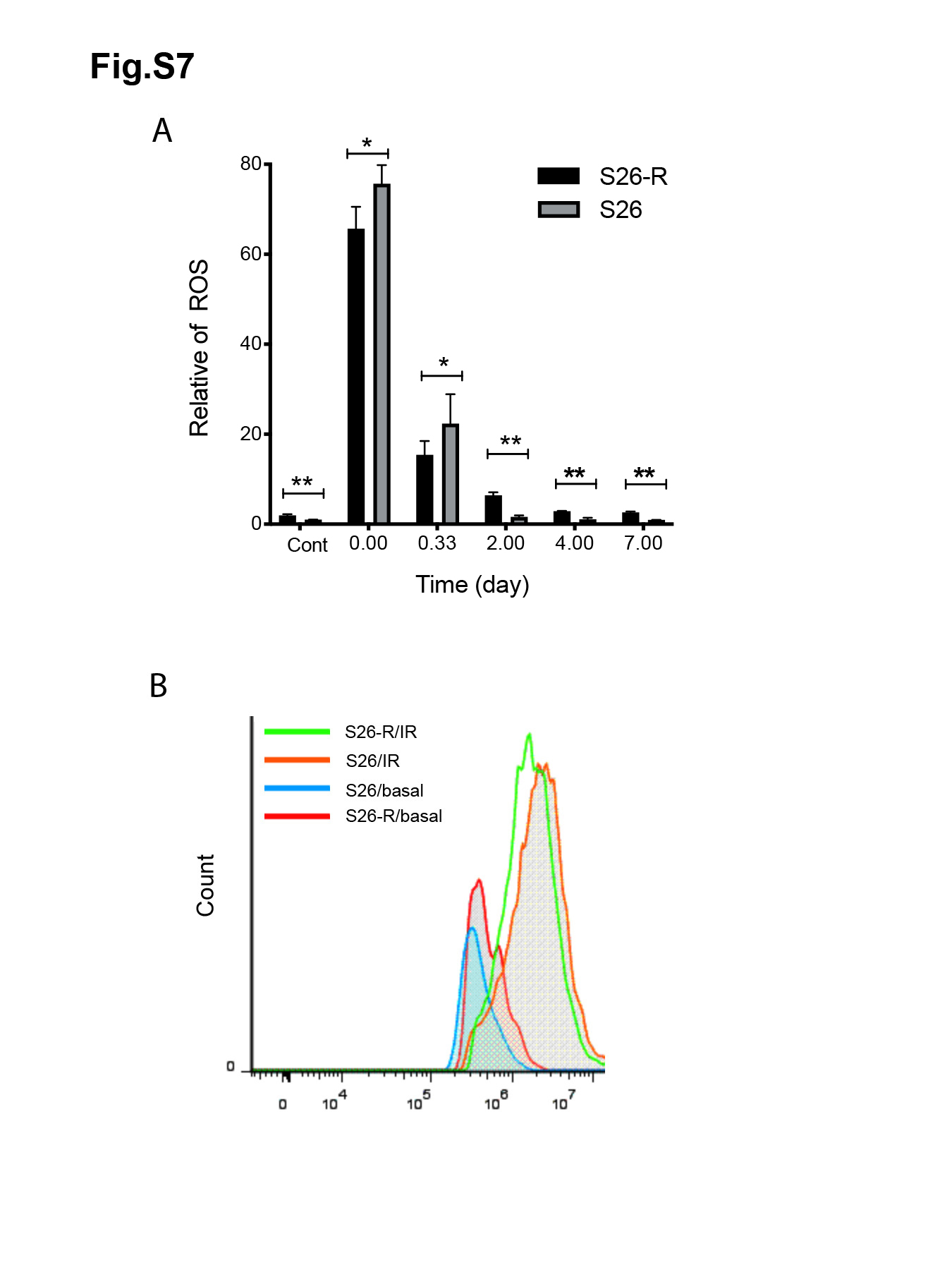

Supplement: Supplementary file 8 — Figure.S7 [file 41419_2022_4551_MOESM8_ESM.jpg]

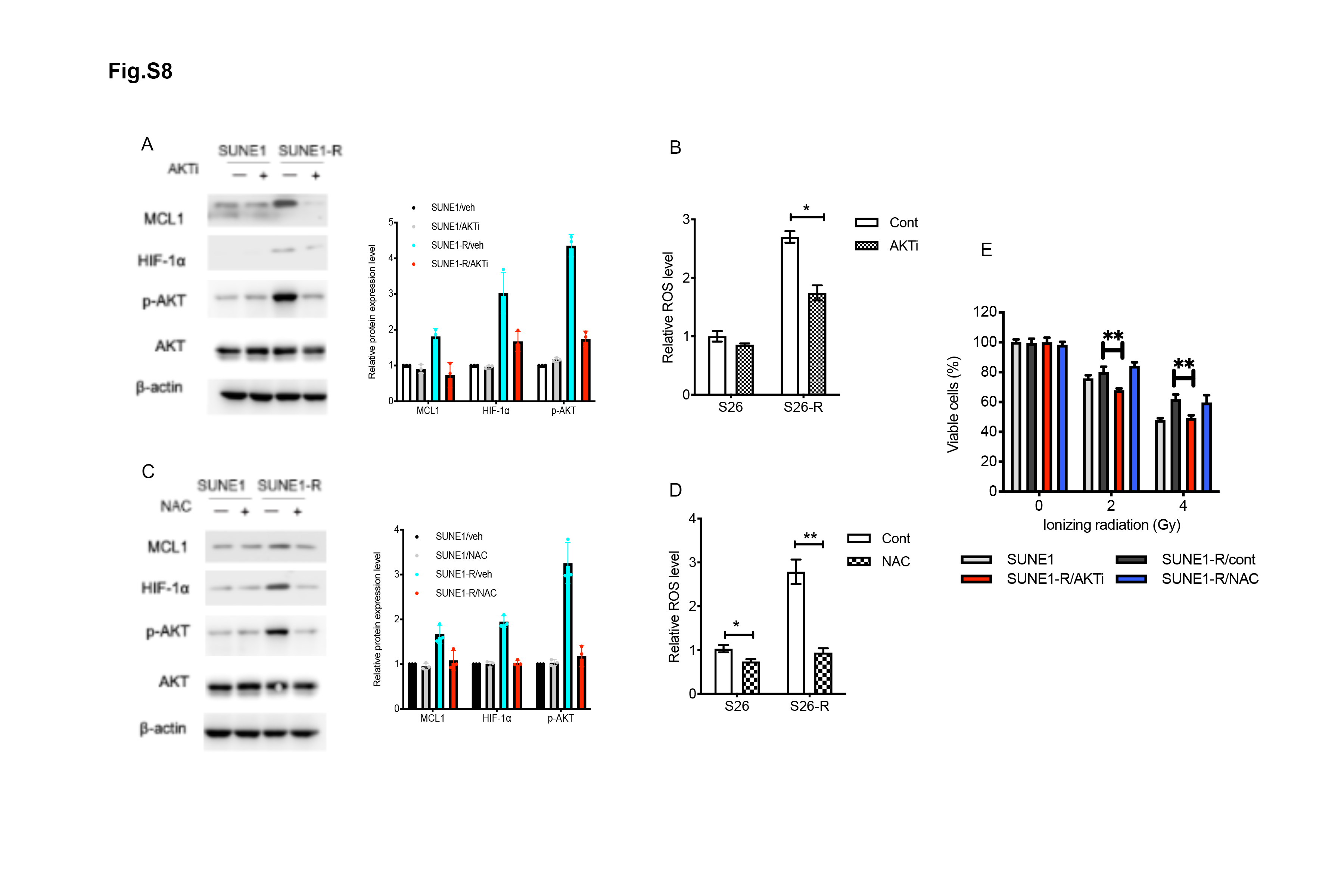

Supplement: Supplementary file 9 — Figure.S8 [file 41419_2022_4551_MOESM9_ESM.jpg]
