# Supplementary material for: Increased MCL-1 synthesis promotes irradiation-induced nasopharyngeal carcinoma radioresistance via regulation of the ROS/AKT loop
Source: Cell Death Dis. 2022 Feb 8;13(2):131. doi: 10.1038/s41419-022-04551-z (PMC8827103; doi:10.1038/s41419-022-04551-z)
Supplement: Supplementary file 11 — agreement of co-authors [file 41419_2022_4551_MOESM11_ESM.pdf]

Re:Adding co-authors in our manuscript

发件人：mozhiwen308 <mozhiwen308@163.com> 自动归档
时　间：2022年1月10日(星期一) 上午8:30
收件人：袁亚维 <yuanyawei@gzhmu.edu.cn>

翻译全文 |

I agree with the changes.
Zhiwen Mo.

At 2022-01-09 22:09:53, "袁亚维" <yuanyawei@gzhmu.edu.cn> wrote:

> Dear all,
> Chun-shan Liu and Huiping Liang would be listed as co-authors in our submitted manuscript after peer-review as they helped to provide important research data for review. Please feel free to contact me by email if you have any questions.
> Best Regards,
> Yawei

回复:Adding co-authors in our manuscript

发件人：Yingying Liang <2015684002@gzhmu.edu.cn>
时　间：2022年1月9日(星期天) 晚上10:11
收件人：袁亚维 <yuanyawei@gzhmu.edu.cn>

翻译全文 |

Dear Yawei,
I agree with the changes.
Yingying
--------------原始邮件--------------
发件人："袁亚维 "<yuanyawei@gzhmu.edu.cn>;
发送时间：2022年1月9日(星期天) 晚上10:09
收件人："yingyingliang" <yingyingliang@gzhmu.edu.cn>;"jianglili" <jianglili@gzhmu.edu.cn>;"shaoxunfan" <shaoxunfan@hotmail.com>;"mozhiwen308" <mozhiwen308@163.com>;"18302078099" <18302078099@163.com>;"xuanan" <xuanan@gzhmu.edu.cn>;"001397" <001397@sina.com.cn>;"1105600015" <1105600015@qq.com>;"lianghuiping" <lianghuiping@gzhmu.edu.cn>;
主题：Adding co-authors in our manuscript

Dear all,
Chun-shan Liu and Huiping Liang would be listed as co-authors in our submitted manuscript after peer-review as they helped to provide important research data for review. Please feel free to contact me by email if you have any questions.
Best Regards,
Yawei

回复：Adding co-authors in our manuscript

发件人：18302078099 <18302078099@163.com> 自动归档
时　间：2022年1月9日(星期天) 晚上10:29
收件人：袁亚维 <yuanyawei@gzhmu.edu.cn>

翻译全文 |

dear yawei
I agree with the changes.
Feiyu Niu

发自我的手机

-------- 原始邮件 --------
发件人： 袁亚维 <yuanyawei@gzhmu.edu.cn>
日期： 2022年1月9日周日 晚上10:09
收件人： yingyingliang <yingyingliang@gzhmu.edu.cn>, jianglili <jianglili@gzhmu.edu.cn>, shaoxunfan <shaoxunfan@hotmail.com>, mozhiwen308 <mozhiwen308@163.com>, 18302078099 <18302078099@163.com>, xuanan <xuanan@gzhmu.edu.cn>, 001397 <001397@sina.com.cn>, 1105600015 <1105600015@qq.com>, lianghuiping <lianghuiping@gzhmu.edu.cn>
主 题： Adding co-authors in our manuscript
Dear all,
Chun-shan Liu and Huiping Liang would be listed as co-authors in our submitted manuscript after peer-review as they helped to provide important research data for review. Please feel free to contact me by email if you have any questions.

Best Regards,

Yawei

Re:Adding co-authors in our manuscript

发件人：徐安安 <xuanan@gzhmu.edu.cn>
时　间：2022年1月9日(星期天) 晚上10:32
收件人：袁亚维 <yuanyawei@gzhmu.edu.cn>

翻译全文 |

Dear Yawei,
I agree with the changes.
Xu anan

------------------ Original ------------------

From: "袁亚维"<yuanyawei@gzhmu.edu.cn>;
Date: Sun, Jan 9, 2022 10:09 PM
To: "yingyingliang"<yingyingliang@gzhmu.edu.cn>; "jianglili"<jianglili@gzhmu.edu.cn>; "shaoxunfan"<shaoxunfan@hotmail.com>; "mozhiwen308"<mozhiwen308@163.com>; "18302078099"<18302078099@163.com>; "徐安安"<xuanan@gzhmu.edu.cn>; "001397"<001397@sina.com.cn>; "1105600015"<1105600015@qq.com>; "lianghuiping"<lianghuiping@gzhmu.edu.cn>;
Subject: Adding co-authors in our manuscript

Dear all,
Chun-shan Liu and Huiping Liang would be listed as co-authors in our submitted manuscript after peer-review as they helped to provide important research data for review. Please feel free to contact me by email if you have any questions.
Best Regards,
Yawei

Dear Yawei,

I agree with the changes.

Lili Jiang

该邮件从移动设备发送

--------------原始邮件--------------
发件人："袁亚维 "<yuanyawei@gzhmu.edu.cn>;
发送时间：2022年1月9日(星期天) 晚上10:09
收件人："yingyingliang" <yingyingliang@gzhmu.edu.cn>;"jianglili" <jianglili@gzhmu.edu.cn>;"shaoxunfan" <shaoxunfan@hotmail.com>;"mozhiwen308" <mozhiwen308@163.com>;"18302078099" <18302078099@163.com>;"xuanan" <xuanan@gzhmu.edu.cn>;"001397" <001397@sina.com.cn>;"1105600015" <1105600015@qq.com>;"lianghuiping" <lianghuiping@gzhmu.edu.cn>;
主题：Adding co-authors in our manuscript

Dear all,
Chun-shan Liu and Huiping Liang would be listed as co-authors in our submitted manuscript after peer-review as they helped to provide important research data for review. Please feel free to contact me by email if you have any questions.
Best Regards,
Yawei

回复：Adding co-authors in our manuscript

发件人：1105600015 <1105600015@qq.com> 自动归档
时　间：2022年1月9日(星期天) 晚上10:29
收件人：袁亚维 <yuanyawei@gzhmu.edu.cn>

翻译全文 |

Dear Yawei,
I agree with the changes.
Chun-shan Liu

------------------ 原始邮件 ------------------

发件人: "袁亚维" <yuanyawei@gzhmu.edu.cn>;
发送时间: 2022年1月9日(星期天) 晚上10:09
收件人: "yingyingliang"<yingyingliang@gzhmu.edu.cn>;"jianglili"<jianglili@gzhmu.edu.cn>;"shaoxunfan"<shaoxunfan@hotmail.com>;"mozhiwen308"<mozhiwen308@163.com>;"18302078099"<18302078099@163.com>;"xuanan"<xuanan@gzhmu.edu.cn>;"001397"<001397@sina.com.cn>;"fly$纶丶sea彡✘"<1105600015@qq.com>;"lianghuiping"<lianghuiping@gzhmu.edu.cn>;
主题: Adding co-authors in our manuscript

Dear all,
Chun-shan Liu and Huiping Liang would be listed as co-authors in our submitted manuscript after peer-review as they helped to provide important research data for review. Please feel free to contact me by email if you have any questions.
Best Regards,
Yawei

Re:Adding co-authors in our manuscript

发件人：梁惠平 <lianghuiping@gzhmu.edu.cn>
时　间：2022年1月9日(星期天) 晚上10:29
收件人：袁亚维 <yuanyawei@gzhmu.edu.cn>

翻译全文 |

Dear Yawei,
I agree with the changes.
Huiping Liang

------------------ Original ------------------

From: "袁亚维"<yuanyawei@gzhmu.edu.cn>;
Date: Sun, Jan 9, 2022 10:09 PM
To: "yingyingliang"<yingyingliang@gzhmu.edu.cn>; "jianglili"<jianglili@gzhmu.edu.cn>; "shaoxunfan"<shaoxunfan@hotmail.com>; "mozhiwen308"<mozhiwen308@163.com>; "18302078099"<18302078099@163.com>; "xuanan"<xuanan@gzhmu.edu.cn>; "001397"<001397@sina.com.cn>; "1105600015"<1105600015@qq.com>; "lianghuiping"<lianghuiping@gzhmu.edu.cn>;
Subject: Adding co-authors in our manuscript

Dear all,
Chun-shan Liu and Huiping Liang would be listed as co-authors in our submitted manuscript after peer-review as they helped to provide important research data for review. Please feel free to contact me by email if you have any questions.
Best Regards,
Yawei

Dear Yawei,
I agree with the changes.
Yu-fan Huang

----- 原始邮件 -----
发件人："袁亚维" <yuanyawei@gzhmu.edu.cn>
收件人："yingyingliang" <yingyingliang@gzhmu.edu.cn>, "jianglili" <jianglili@gzhmu.edu.cn>, "shaoxunfan" <shaoxunfan@hotmail.com>, "mozhiwen308" <mozhiwen308@163.com>, "18302078099" <18302078099@163.com>, "xuanan" <xuanan@gzhmu.edu.cn>, "001397" <001397@sina.com.cn>, "1105600015" <1105600015@qq.com>, "lianghuiping" <lianghuiping@gzhmu.edu.cn>
主题：Adding co-authors in our manuscript
日期：2022年01月09日 22点09分

Dear all,
Chun-shan Liu and Huiping Liang would be listed as co-authors in our submitted manuscript after peer-review as they helped to provide important research data for review. Please feel free to contact me by email if you have any questions.
Best Regards,
Yawei

Re: Adding co-authors in our manuscript

发件人: **dr.shaoxunfan@qq.com** <dr.shaoxunfan@qq.com>
时　间: 2022年1月10日(星期一) 上午9:01
收件人: 袁亚维 <yuanyawei@gzhmu.edu.cn>; 1305224951 <1305224951@qq.com>

翻译全文 |

为了营造绿色健康的邮箱环境，我们想了解一下，这是否您订阅的邮件？　是我订阅的　不是我订阅的　我不确定　自动归档

Dear Dr. Yuan

I agree with the changes.

Xunfan Shao

dr.shaoxunfan@qq.com

From: 袁亚维
Date: 2022-01-09 22:51
To: 1305224951
Subject: Adding co-authors in our manuscript

Dear Dr.Shao,
Chun-shan Liu and Huiping Liang would be listed as co-authors in our submitted manuscript after peer-review as they helped to provide important research data for review. Please feel free to contact me by email if you have any questions.
Best Regards,
Yawei
