# Supplementary material for: Increased MCL-1 synthesis promotes irradiation-induced nasopharyngeal carcinoma radioresistance via regulation of the ROS/AKT loop
Source: Cell Death Dis. 2022 Feb 8;13(2):131. doi: 10.1038/s41419-022-04551-z (PMC8827103; doi:10.1038/s41419-022-04551-z)
Supplement: Supplementary file 12 — author contribution [file 41419_2022_4551_MOESM12_ESM.docx]

**Author contributions**

Y.Y and Z.M guided the whole work. Y.L and Y.Y conceptualized and designed the study. Y.L, F.N and A.X performed most of the experiments. L.J helped with validation and methodology. C.L and H.L helped to add data for review. Z.M and X.S collected and analyzed clinical data with software. Y.L and F.N wrote the manuscript. Y.W helped to revise the manuscript. All authors read and approved the final manuscript.
