# Supplementary material for: Increased MCL-1 synthesis promotes irradiation-induced nasopharyngeal carcinoma radioresistance via regulation of the ROS/AKT loop
Source: Cell Death Dis. 2022 Feb 8;13(2):131. doi: 10.1038/s41419-022-04551-z (PMC8827103; doi:10.1038/s41419-022-04551-z)
Supplement: Supplementary file 13 — conflict of interest [file 41419_2022_4551_MOESM13_ESM.docx]

**Conflict of interest statement**

# We declare that we have no financial and personal relationships with other people or organizations that can inappropriately influence our work, there is no professional or other personal interest of any nature or kind in any product, service or company that could be construed as influencing the position presented in our manuscript entitled “Increased MCL-1 Synthesis Promotes Irradiation-induced Nasopharyngeal Carcinoma Radioresistance *via* Regulation of the ROS/AKT loop”.
